# G2385R and I2020T Mutations Increase LRRK2 GTPase Activity

**DOI:** 10.1155/2016/7917128

**Published:** 2016-05-25

**Authors:** Dong Hwan Ho, Jihoon Jang, Eun-hye Joe, Ilhong Son, Hyemyung Seo, Wongi Seol

**Affiliations:** ^1^InAm Neuroscience Research Center, Sanbon Medical Center, College of Medicine, Wonkwang University, Sanbondong, Gunposhi, Gyeonggido 15865, Republic of Korea; ^2^Department of Molecular and Life Sciences, Hanyang University, Ansanshi, Gyeonggido 15588, Republic of Korea; ^3^Department of Pharmacology, Ajou University School of Medicine, Suwonshi 16499, Republic of Korea; ^4^Neuroscience Graduate Program, Ajou University School of Medicine, Suwonshi 16499, Republic of Korea; ^5^Institute for Medical Sciences, Ajou University School of Medicine, Suwonshi 16499, Republic of Korea; ^6^Department of Neurology, Sanbon Medical Center, College of Medicine, Wonkwang University, Sanbondong, Gunposhi, Gyeonggido 15865, Republic of Korea

## Abstract

The LRRK2 mutation is a major causal mutation in familial Parkinson's disease. Although LRRK2 contains functional GTPase and kinase domains and their activities are altered by pathogenic mutations, most studies focused on LRRK2 kinase activity because the most prevalent mutant, G2019S, enhances kinase activity. However, the G2019S mutation is extremely rare in the Asian population. Instead, the G2385R mutation was reported as a major risk factor in the Asian population. Similar to other LRRK2 studies, G2385R studies have also focused on kinase activity. Here, we investigated GTPase activities of G2385R with other LRRK2 mutants, such as G2019S, R1441C, and I2020T, as well as wild type (WT). Our results suggest that both I2020T and G2385R contain GTPase activities stronger than that of WT. A kinase assay using the commercial recombinant proteins showed that I2020T harbored stronger activity, whereas G2385R had weaker activity than that of WT, as reported previously. This is the first report of LRRK2 I2020T and G2385R GTPase activities and shows that most of the LRRK2 mutations that are pathogenic or a risk factor altered either kinase or GTPase activity, suggesting that their physiological consequences are caused by altered enzyme activities.

## 1. Introduction

Leucine-rich repeat kinase 2 (LRRK2) was identified as a gene corresponding to the Park8 locus in 2004 [1, 2] and now recognized as the gene with the most prevalent mutation in familial Parkinson's disease (PD, [3]). LRRK2 has been intensively studied to elucidate the pathogenic mechanism of PD [4–6]. In addition to *α*-synuclein that is a Lewy body component, LRRK2 has emerged as a major therapeutic target for PD because it contains two active enzyme functions regulating cellular signals, kinase, and GTPase, whose activities are theoretically modulated by small therapeutic molecules. In particular, the most prevalent pathogenic G2019S mutation accounts for up to 40% of familial and sporadic PD cases depending on ethnic background [7, 8] and significantly increases its kinase activity [9, 10]. The result that the G2019S mutant reportedly displays increased kinase activity compared to that of the wild type (WT) leads to the idea that a LRRK2 kinase inhibitor could be a promising PD therapeutic drug [11]. Actually, considerable efforts have been focused on developing pharmaceutically active LRRK2 kinase inhibitors. However, the GTPase function of LRRK2 has been less studied, except in R1441G/H/C mutants [12]. Therefore, we measured the GTPase activities of various LRRK2 mutants and the WT. In particular we included G2385R in our assay. Although the G2019S mutation exhibits increased kinase activity, it is rarely found in the Asian population [13] unlike the European population [7]. In contrast, the G2385R mutation has been reported as a risk factor for PD in Asians including Chinese Han from Singapore and Taiwan [14–16], Japan [17], and Korea [18]. Interestingly, the G2385R mutation is not found in Caucasians [19]. The G2019S mutation resides in its kinase domain, whereas the G2385R mutation is present in the WD40 domain, which is one of two protein-protein interaction domains in LRRK2 along with the LRR domain. A WD40 domain consists of approximately 40 amino acids and seven *β*-blades and its major function is to form multiple protein complexes. In addition, experimental evidence for the WD40 domain in the LRRK2 protein has been reported by electron microscopy using the C-terminal of the LRRK2 protein [20]. In addition, G2385R has been reported to reduce interactions between LRRK2 WD40 and synaptic vesicles [20].

In contrast to the G2019S mutation, the G2385R mutation has been less studied, and the G2385R kinase activity results are controversial (Table 1, [21–23]). In addition, GTPase activity of G2385R has not been investigated, although its GTP binding activity is similar to that of WT [21].

I2020T is another pathogenic mutant located in the kinase domain along with G2019S. Although the relative kinase activity level of I2020T has been reported to increase compared to that of WT, it remains controversial like G2385R (Table 1, [24, 25]). The issue of whether the pathogenic LRRK2 mutations enhance their kinase activities is critical for a strategy to develop LRRK2 kinase inhibitor as PD therapeutics. Therefore, we tested kinase and GTPase activities of I2020T and G2385R along with other LRRK2 mutants using commercial recombinant proteins and immunoprecipitates (IP) of proteins transiently expressed in HEK 293T cells.

## 2. Materials and Methods

### 2.1. Plasmids, Proteins, and Antibodies

The myc-tagged LRRK2 WT, G2019S, R1441C, D1994A, and G2385R plasmids were described previously [26, 27]. The GST-ΔN (950-2527) LRRK2 WT, G2019S, R1441C, and D1994A recombinant proteins were purchased from Invitrogen (Carlsbad, CA, USA) and GST-ΔN LRRK2 I2020T and G2385R were obtained from Creative Biomart (Shirley, NY, USA). Antibodies were purchased and used as follows. Monoclonal anti-LRRK2 (MJFF2; 1 : 10000 for recombinant LRRK2 or 1 : 1000 for HEK 293T cell lysates) and anti-pS935 LRRK2 (UDD2 10 [12], 1 : 1000) were obtained from Abcam (Cambridge, MA, USA); monoclonal anti-myc (9E10 and 9E11; 1 : 2000) and polyclonal anti-Rab5B (C-12; 1 : 500) were from Santa Cruz Biotechnology (Santa Cruz, CA, USA); polyclonal phospho-threonine-X-arginine antibody (2351S; 1 : 1000), monoclonal phospho-AKT at ser473 (193H12, 1 : 1000), and polyclonal anti-AKT (9272; 1 : 1000) were purchased from Cell Signaling Technology (Danvers, MA, USA).

### 2.2. *In Vitro* Kinase and GTPase Assays

We used recombinant GST-ΔN LRRK2 protein (Invitrogen) and His-Rab5B purified from the* E. coli* BL21 strain for the* in vitro* LRRK2 kinase assay. The indicated proteins (100 ng) were incubated in 20 *μ*L kinase buffer [25 mM Tris-HCl (pH 7.5), 5 mM *β*-glycerolphosphate, 2 mM DTT, 0.1 mM NA_3_VO_4_, 10 mM MgCl_2_, and 5 *μ*Ci [*γ*-^32^P] ATP (BLU502; Perkin Elmer, Waltham, MA, USA) or 50 *μ*M ATP] at 33°C for 30 min. The samples were subjected to sodium dodecyl sulfate-polyacrylamide gel electrophoresis, and the dried gel was analyzed by autoradiography or transferred to a nitrocellulose membrane for Western blotting.

The GTPase assay was carried out using a commercial GTPase assay kit (602-0120; Innova Bioscience, Cambridge, UK). One *μ*g of GST-ΔN LRRK2 or the LRRK2 IP were used for each GTPase assay. We performed the assay and calculated enzyme activity as recommended by the manufacturer's instructions. The optical density (OD) value of the buffer (*in vitro* assay) or vector (IP samples) was subtracted from the OD value for LRRK2 GTPase activity and interpolated with a standard curve. The quantity of LRRK2 proteins used in each assay was estimated by applying densitometry software (Multi Gauge V3.0; Fuji, Tokyo, Japan) to the Western blot. The interpolated values were divided by quantity of LRRK2 protein for normalization.

### 2.3. Transfection, Immunoprecipitation, and Western Blot Analysis

Human HEK 293T cells (2 × 10^6^) were plated in 100 mM dishes (SPL, Gyeonggido, Republic of Korea). The cells were cultured at 37°C in a 5% CO_2_ incubator in the DMEM medium with 10% fetal bovine serum (Corning, Manassas, VA, USA) and 1x antibiotic antimycotic solution (Thermo, Waltham, MA, USA). The media was exchanged 30 min before transfection. The indicated LRRK2 DNAs (5 *μ*g) were transfected with Lipofectamine 2000 (Invitrogen). The day after transfection, the cells were lysed with PBS, 1% Triton-X100, 1x PhosSTOP (Roche, Basal, Switzerland), and 1x Xpert-PIC (GenDEPOT, Barker, TX, USA). The cell lysate supernatant was obtained by centrifugation at 16,000 ×g for 10 min at 4°C and was incubated with 2 *μ*g myc (9E10) antibody and protein-A agarose (Pierce, Rockford, IL, USA) for 18 hr at 4°C. After a brief centrifugation and repeated washing, the IP were subjected to Western blotting. HEK 293T whole cell lysates were also used for Western blotting to detect the protein of interest using the indicated antibodies.

### 2.4. Statistical Analysis

All statistical analyses were performed with Prism 6 program software (GraphPad Software, La Jolla, CA, USA). All data sets were analyzed by one-way analysis of variance (ANOVA) followed by Tukey's multiple comparisons test. A *p* value < 0.05 was considered significant.

## 3. Results and Discussion

### 3.1. G2385R and I2020T Increase GTPase Activity

**Figure 1:**
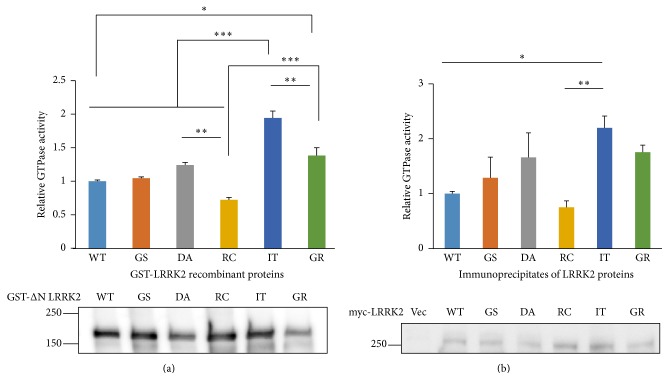
GTPase assays of various LRRK2 proteins. (a) GTPase assays of the commercially available recombinant LRRK2 proteins. (b) GTPase assays using the immunoprecipitates of myc-tagged LRRK2 proteins transiently expressed in HEK 293T cells. The assay was carried out using a commercial GTPase assay kit. The relative GTPase activity results are shown after a statistical analysis of three independent experiments (top). Each GTPase activity value was divided by the quantity of protein used in the assay measured using GST (a) or myc (b) antibodies. Representative Western blots were shown (bottom). All values are relative to the WT and are expressed as the mean ± SEM. Vec: empty vector, WT: wild type, GS: G2019S, DA: D1994A, RC: R1441C, IT: I2020T, and GR: G2385R. ^*∗*^ *p* ≤ 0.05, ^*∗∗*^ *p* ≤ 0.01, and ^*∗∗∗*^ *p* ≤ 0.001.

We used five commercial LRRK2 mutant proteins, such as G2019S, R1441C, I2020T, D1994A, and G2385R, with the WT proteins. They all were GST proteins fused to the LRRK2 whose N-terminal 969 amino acids were deleted. The recombinant proteins were tested in the GTPase assays, and the results are shown in Figure 1(a). Surprisingly, I2020T showed the highest GTPase activity (twofold increase compared to that of the WT) which was significantly higher than that of all other LRRK2 proteins tested. The G2019S shows little difference from the WT as previously described [28, 29]. In addition, G2385R also showed a significant increase compared to that of WT and R1441C. The R1441C mutant showed slight decreased activity, as reported previously [28–30]. Interestingly, the kinase-dead D1994A mutant exhibited significantly higher activity than that of the R1441C mutant (Figure 1(a)).

To investigate this result further, we used the IP of LRRK2 proteins transiently expressed in HEK 293T cells for the GTPase assays. We assumed that the LRRK2 protein IP might contain its binding proteins and maintain the cellular complex; thus, they may be better representatives of the physiological LRRK2 complex compared to the recombinant proteins purified from insect Sf9 cells. The GTPase assay using the IP showed a pattern similar to that of the recombinant proteins, confirming that I2020T exhibited the highest GTPase activity among the LRRK2 proteins tested, and R1441C had lower activity than that of WT (Figure 1(b)).

The GTPase activities of the LRRK2 mutants were investigated mainly with R1441C/G, whose mutation is present in the GTPase domain, which impairs GTPase activity [30, 31]. No study has evaluated GTPase activities of G2385R or I2020T, although an early report studied the GTP binding activity of the most well-known LRRK2 mutants. The results showed that the R1441C/G, I1371V, and Y1699C mutants, with mutations present in the GTPase/ROC domain, have higher GTP binding activity than that of WT [21]. However, both G2385R and I2020T show GTP binding activity similar to that of WT [21]. Taken together, our results suggest that the GTPases of both G2385R and I2020T have a weak GTP binding signal because their active GTP-bound status might be shorter than that of WT.

G2385 is located on the surface of the WD40 domain, which is one of the protein-protein interaction domains. An additional positive charge in the G2385R domain may alter intramolecular and/or intermolecular interactions of LRRK2 [22], resulting in a change in its GTPase activity. In addition, G2385 is located in one of 25 LRRK2 myristoylation candidate sites, suggesting that LRRK2 G2385R may be relocated from the membrane to the lumen of the microsome where G2385R is mainly present [22]. One study reported that SAR1 GTPase activity remodels lipid bilayers and affects membrane fission [32]. As LRRK2 is present in the membranes of various organelles [33] and regulates synaptic vesicle endocytosis [26], it would be interesting to study whether G2385R differentially regulates endocytosis compared to that of WT.

### 3.2. I2020T Increases Kinase Activity, Whereas G2385R Decreases Kinase Activity

Because the most prevalent pathogenic G2019S mutant increased its kinase activity, kinase activity of the LRRK2 mutants, particularly that of G2019S, was actively investigated. However, kinase studies with G2385R and I2020T are relatively rare and showed contradictory results (Table 1).

Most LRRK2 mutant kinase assays have been analyzed based on LRRK2 autophosphorylation or LRRKtide, a peptide containing the LRRK2 phosphorylated motif or MBP, the general nonspecific kinase substrate, and most studies used radiolabeled isotope for detection (Table 1). However, we used Rab5B, LRRK2 kinase substrate [37] in our LRRK2 kinase assay, in addition to its autophosphorylation function.

**Figure 2 fig2:**
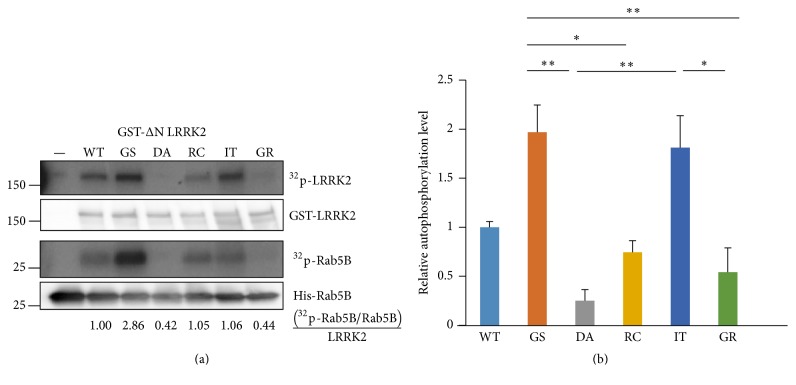
*In vitro* kinase assays with commercially available recombinant LRRK2 proteins. The assays were carried out using purified recombinant His-Rab5B protein as the substrate. (a) LRRK2 and Rab5B phosphorylations were shown by autoradiography. The quantities of the LRRK2 and Rab5B proteins used are shown in the Western blot results with the GST and Rab5B antibodies below the corresponding autoradiograms. Relative Rab5B phosphorylation was calculated and is shown below the Rab5B Western blot. (b) Relative kinase activities of autophosphorylated LRRK2 after a statistical analysis of three independent experiments. All values are relative to the WT and are expressed as the mean ± SEM. —: no added LRRK2 protein, WT: wild type, GS: G2019S, DA: D1994A, RC: R1441C, IT: I2020T, and GR: G2385R. ^*∗*^ *p* ≤ 0.05 and ^*∗∗*^ *p* ≤ 0.01.

The* in vitro* kinase assay showed that LRRK2 autophosphorylation activities were in the order of G2019S > I2020T > R1441C ≥ WT > G2385R (Figure 2(a)). The Rab5B phosphorylation pattern was similar to that of the autophosphorylation although G2019S and I2020T exhibited more and less signals than those of autophosphorylation, respectively (Figure 2(a)). This result suggests that the mutants alter their substrate specificity. Actually, such a possibility was observed in a study reporting that both G2385R and I2020T phosphorylate the T72 residue in Rab8a and the T73 residue in Rab10 more strongly than those of G2019S but that the Rab12 S106 residue is similar to or less than that of the G2019S [38]. This may be due to decreased interactions between G2019S and different Rab proteins, such as AKT1, whose lower phosphorylation by G2019S has been reported to be due to weaker interaction between G2019S and AKT1 [39]. More careful experiments are required.

Two previous studies in Table 1 have reported that I2020T exhibited weaker kinase activity than that of the WT [24, 34] whereas our and other studies showed higher kinase activity than that of the WT (Table 1). Careful examination of the references revealed that Doggett et al. used phospho-LRRK2 antibodies such as S910 or S935 for detection [34] and Jaleel et al. used GST-ΔN LRRK2 [1326–2527] instead of full length protein [24]. Use of the deleted fusion protein [24] could not be the cause of this discrepancy because we observed increase of I2020T kinase activity with similar GST-ΔN LRRK2 (950-2527) LRRK2 proteins. Instead, the use of phospho-S910/935 antibody might be a partial cause of the difference because the antibody can detect only the specified phosphorylated sites whereas the isotope can detect all phosphorylated sites.

G2385R showed decreased kinase activity for both recombinant and immunoprecipitates of proteins (Figure 2(a)) but slightly higher activity than that of the kinase-dead D1994A mutants with no detectable activity, as reported previously [22, 24]. This result was confirmed by the LRRK2 autophosphorylation statistical analysis (Figure 2(b)).

**Figure 3:**
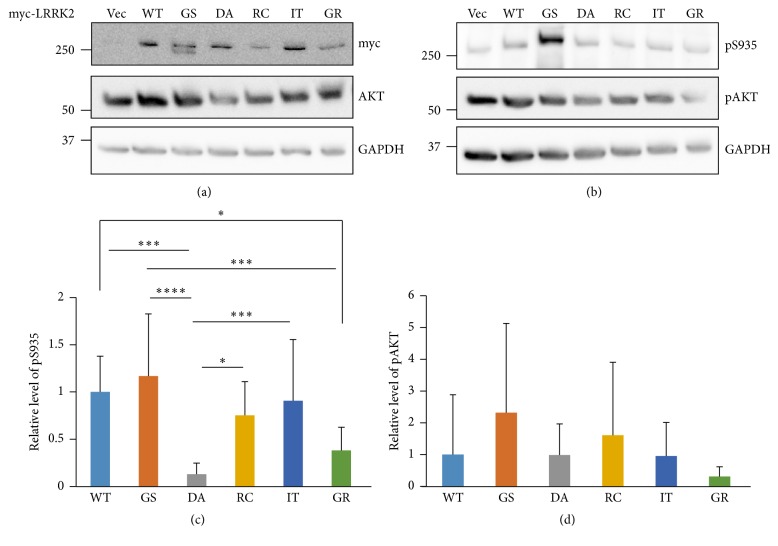
Kinase activities of cellular LRRK2 proteins. HEK 293T cell lysates transiently expressing the indicated myc-tagged LRRK2 WT and mutant proteins were separately loaded onto two gels and analyzed by SDS-PAGE. The membranes were cut to a proper size to detect the corresponding proteins. Myc-tagged LRRK2 and AKT1 were detected by myc and AKT antibodies, respectively ((a): myc and AKT). LRRK2 kinase activities are shown as autophosphorylation of LRRK2 (detected by pS935 antibody, (b): pS935) and phosphorylation of the AKT LRRK2 substrate ((b): pAKT). GAPDH was used as a loading control. A representative of 12 different experiments is shown (a and b). Relative kinase activities of autophosphorylated LRRK2 (c) and phospho-AKT (d) after a statistical analysis were also shown. Relative pS935 or pAKT levels were calculated by dividing the GAPDH value by that for the phosphorylated proteins, which was again divided by total LRRK2 or AKT, respectively. The resulting pAKT number was divided by total LRRK2 (myc) to consider equal quantities of the LRRK2 kinase and substrates (d). All values are relative to the WT and are expressed as the mean ± SD. Vec: empty vector, WT: wild type, GS: G2019S, DA: D1994A, RC: R1441C, IT: I2020T, and GR: G2385R. ^*∗*^ *p* ≤ 0.05, ^*∗∗∗*^ *p* ≤ 0.001, and ^*∗∗∗∗*^ *p* ≤ 0.0001.

We also measured cellular kinase activities of various LRRK2 mutants and WT (Figure 3). AKT1 has been reported as a LRRK2 kinase substrate with its S473 phosphorylation site [39]. Therefore, AKT1 and phosphorylated AKT1 levels were utilized to measure LRRK2 kinase activity with AKT1 and pS473-AKT1 antibodies. We observed no difference in pAKT1 levels among the mutants and WT, although repeated experiments revealed significant differences in the LRRK2 pS935 level among the WT and mutants as previously reported [38]. No difference in pAKT1 levels may be due to other endogenous kinases that phosphorylate the same AKT1 site [40, 41] and may overcome effect of LRRK2 proteins exogenously expressed.

### 3.3. Relationship between LRRK2 GTPase and Kinase Activities

The initial LRRK2 studies suggested that LRRK2 GTPase activity regulates its kinase activity because mutants such as K1347A in which GTP binding activity was impaired showed little kinase activity and incubating GTP with LRRK2 increased its kinase activity [21]. However, a further study suggested that GTP binding capacity, not direct GTP binding itself, was required for LRRK2 kinase activity and incubating GTP with LRRK2 exhibits a modest effect on its kinase activity [42]. Another report with the R1398L mutant, which is unable to hydrolyze GTP, demonstrated that GTP hydrolysis is necessary for LRRK2 kinase activity [43]. In addition, another more recent study suggested that LRRK2 kinase activity enhanced its GTPase activity [44]. However, our data showing that the kinase-dead D1994A exhibited higher GTPase activity than that of the R1441C (Figure 1(a)) suggested more complex regulation of both enzyme functions. Taken together, these results suggest that LRRK2 GTPase and kinase activities regulate each other and that LRRK2 GTPase is different from the classical simple GTPase in which simple GTP binding status regulates the cellular signal as an on-off switch. In our study, G2385R exhibited higher GTPase activity than that of WT but lower kinase activity both* in vitro* and in cellular assays (Figures 1 [Fig fig2]–3), suggesting that the G2385R mutation in the WD40 domain may regulate GTPase function. This could be related to LRRK2 dimerization activity because the WD40 domain functions during dimerization [45] and dimerization is critical for GTPase and kinase activities [12]. Actually, size-exclusion chromatography with various LRRK2 mutants showed that G2385R was eluted at a higher molecular weight mass than that of WT [22]. However, any association between LRRK2 dimerization and GTPase activity should be cautious because another I2020T mutant exhibiting higher GTPase activity has been reported not to exhibit stronger dimerization compared to that of WT [25], and one study reported that LRRK2 is predominantly present as a monomer [46]. I2020T mutant has been reported to be more sensitive to degradation than WT [47] and its instability was suggested as a reason for its neurotoxicity [48]. Deactivation of certain GTPase, Rac, has been reported to be susceptible to its degradation via ubiquitin/proteasome pathway [49]. It may be possible that the increase of GTPase activity of the I2020T changes its ability to recruit downstream target proteins and causes different status of binding proteins related ubiquitin/proteasome pathway.

Both G2385R and I2020T mutations increased GTPase activity, but only G2385R present in WD40 exhibited lower kinase activity than that of WT, suggesting that the G2385R mutation changes the intramolecular interactions between the various LRRK2 domains and the WD40 domain and/or binding status of various LRRK2 interacting partners, critical for its kinase activity. One report to study the WD40 domain structure* in silico* suggested that G2385R substitution causes potential structural change, which may affect interactions of WD40 domain with other proteins [20]. In fact, the same study showed that the LRRK2 WD40 G2385R impairs its binding to synaptic vesicles compared to the LRRK2 WD40 [20]. The structural change of the WD40 G2385R domain may induce conformation change in the GTPase domain and result in changes of its activity and/or interaction pattern with other cellular proteins. The I2020T mutation has been reported to increase tau phosphorylation in cells overexpressing I2020T and tau proteins [50] and to be more sensitive for its degradation [48]. This might be due to the higher GTPase activity which results in a weaker signal to its downstream target and/or change of interactions with other cellular proteins.

## 4. Conclusions

Our results show that the G2385R and I2020T mutants increase their GTPase activities compared to WT and G2019S, suggesting that these activities differentially regulate cellular signals from that of WT and G2019S, and result in different pathophysiological consequences.

## Figures and Tables

**Table 1:** The kinase activities of LRRK2 I2020T and G2385R mutants^*∗*^.

WT^*∗∗*^	I2020T	G2385R	Substrate	Detection	Reference
++	++++	++	LRRK2	Isotope	[21]
++	+++	nt	LRRK2	Isotope	[25]
++	nt	+	LRRK2	Isotope	[22]
++	nt	++	LRRK2, MBP	Isotope	[23]
++	+	1/2+	LRRK2, MBP, moesin	Isotope	[24]
++	1/2+	+	LRRK2	Antibody	[34]
++	+++	nt	LRRK2, 4E-BP1	Isotope	[35]
++	++	++	LRRKtide	Isotope	[36]
++	+++	+	LRRK2, Rab5B	Isotope	This study

+: assigned based on the graphic values of each mutant from the indicated studies.

nt: not tested.

^*∗*^All previous experiments cited here have used immunoprecipitates of cell lysate that was transiently transfected with full length LRRK2 except [24] which has used GST-LRRK2 [1326–2527] for transfection. The kinase activities have been detected by either [*γ*-^32^P] ATP or phospho-LRRK2 antibodies such as pS910 or pS935.

^*∗∗*^The WT activity of each study was arbitrarily assigned as ++ and compared within each study, not between studies.
